# EGR1 mediates miR-203a suppress the hepatocellular carcinoma cells progression by targeting HOXD3 through EGFR signaling pathway

**DOI:** 10.18632/oncotarget.9605

**Published:** 2016-05-26

**Authors:** Lumin Wang, Hongfei Sun, Xiaofei Wang, Ni Hou, Lingyu Zhao, Dongdong Tong, Kang He, Yang Yang, Tusheng Song, Jun Yang, Chen Huang

**Affiliations:** ^1^ Department of Cell Biology and Genetics, School of Basic Medical Sciences, Xi'an Jiaotong University Health Science Center, Xi'an, Shaanxi, P.R. China; ^2^ Key Laboratory of Environment and Genes Related to Diseases, Xi'an Jiaotong University Health Science Center, Xi'an, Shaanxi, P.R. China; ^3^ Department of Pathology, Second Affiliated Hospital of Xi'an Jiaotong University College of Medicine, Xi'an, Shaanxi, P.R. China; ^4^ Cardiovascular Research Center, Xi'an Jiaotong University Health Science Center, Xi'an, Shaanxi, P.R. China

**Keywords:** EGR1, miR-203a, HOXD3, hepatocellular carcinoma, cell progression

## Abstract

EGR1 plays a critical role in cancer progression. However, its precise role in hepatocellular carcinoma has not been elucidated. In this study, we found that the overexpression of EGR1 suppresses hepatocellular carcinoma cell proliferation and increases cell apoptosis by binding to the miR-203a promoter sequence. In addition, we investigated the function of miR-203a on progression of HCC cells. We verified that the effect of overexpression of miR-203a is consistent with that of EGR1 in regulation of cell progression. Through bioinformatic analysis and luciferase assays, we confirmed that miR-203a targets HOXD3. Silencing HOXD3 could block transition of the G2/M phase, increase cell apoptosis, decrease the expression of cell cycle and apoptosis-related proteins, EGFR, p-AKT, p-ERK, CCNB1, CDK1 and Bcl2 by targeting EGFR through EGFR/AKT and ERK cell signaling pathways. Likewise, restoration of HOXD3 counteracted the effects of miR-203a expression.

In conclusion, our findings are the first to demonstrate that EGR1 is a key player in the transcriptional control of miR-203a, and that miR-203a acts as an anti-oncogene to suppress HCC tumorigenesis by targeting HOXD3 through EGFR-related cell signaling pathways.

## INTRODUCTION

Hepatocellular carcinoma (HCC) is the sixth most common malignant cancer and the third leading cause of cancer-related death worldwide [1, 2]. Although the survival of patients with HCC has improved because of advances in surgical resection and peri-operative management, uncontrolled tumor metastasis, frequent intrahepatic spread, and extrahepatic metastasis are the primary causes of low short-term survival rates after surgical resection [3]. Therefore, a better understanding of the molecular mechanisms underlying HCC progression remains essential for the development of new therapeutic strategies.

EGR1 is a member of the early growth response (EGR) family of transcription factors that also includes EGR2, EGR3, and EGR4. These four proteins have a high degree of homology at their DNA-binding zinc finger domains, which bind to a GC-rich fragment in the promoter region of their target genes [4]. Aberrant EGR1 expression is linked to human diseases such as ischemic injury, cancer, inflammation, atherosclerosis, and cardiovascular pathogenesis [5]. The function of EGR1 in cancer pathogenesis is complex because it functions both in-cell proliferation and apoptosis. In some cancers, EGR1 is associated with tumor progression, for instance in prostate and gastric cancer, which possess endocrine components [6, 7], whereas in others, EGR1 exhibits prominent tumor-suppressive activity by activating major tumor suppressor factors, including transforming growth factor-β1, p53, p73, fibronectin, and PTEN. However, the relationship between EGR1 and miR-203a has yet to be clarified.

MicroRNAs (miRNAs) are a class of small, non-coding, single-stranded RNAs that play important roles in the pathogenesis of human diseases by modulating the activity of specific mRNA targets. They regulate a wide range of biological processes, including cell proliferation and differentiation, migration, apoptosis, development and metabolism by targeting 3′-untranslated regions (3′-UTRs) of mRNAs [8, 9]. Several studies have suggested that deregulation of miRNAs may be associated with cancer initiation and development, for example: miR-29c in gastric cancer [10], miR-135 in breast cancer [11], and the miR-179~2 cluster in non-small cell lung cancer [12]. These findings underscore the need for in-depth searches for miRNAs that are aberrantly expressed during carcinogenesis, as well as intensive investigation of their roles in tumor biology.

The miRNA, miR-203, is located on chromosome 14q32.33 and has been reported as a tumor suppressor involved in the proliferation, apoptosis, invasion, and migration of cancer cells [13–15]. miR-203 can suppress the development of oral cancer by downregulating Yes-1 and inhibiting the proliferation and migration of lung cancer cells by targeting PKCα [16]. In addition, miR-203 suppresses the proliferation and migration of cells through the Robo/ERK/MMP-9 pathway [17]. However, the mechanism of miR-203 regulation in HCCs has not yet been studied.

Numerous genes are associated with carcinogenesis and play regulatory roles in tumor promotion and maintenance [18, 19]. Several studies have demonstrated that the clustered homeobox (HOX) genes are important factors in cancer pathogenesis. Numerous examples of deregulated HOX expression have been found in solid tumors, such as acute myeloid leukemia [20], head and neck squamous cell carcinoma [21], and cervical cancer [22]. Deregulation of HOX genes influences tumorigenesis and cancer cell biology through differentiation, apoptosis, receptor signaling, and other unknown mechanisms [23]. HOXD3 is a third paralogous member of the HOXD gene family, and plays a role in the formation of somatic mesoderm- and neural crest-derived structures during mouse development [24, 25]. The function of HOXD3 in the regulation of immigration or invasion-related gene expression has been investigated. Overexpression of the HOXD3 gene results in increased integrin-alpha-v-beta-3 expression and decreased E-cadherin levels in A549 cells [26]. Furthermore, transduction of HOXD3-antisense oligos into human melanoma cells results in decreased invasive and motile activity [27]. However, little is known about the mechanisms by which HOXD3 affects tumor proliferation and apoptosis, especially in HCC.

Herein, we provide evidence that EGR1 mediates miR-203a, and plays a crucial role in the regulation of proliferation and apoptosis of HCC cells via HOXD3-related EGFR/AKT or ERK cell signaling pathways. Our results are based on gain- and loss-of-function studies of EGR1, miR-203a and HOXD3 *in vivo* and *in vitro*. To our knowledge, this is the first study that directly illustrates a correlation between EGR1 and miR-203a, miR-203a and HOXD3, HOXD3 and EGFR. Thus, the EGR1-miR-203a interaction may serve as a potential prognostic marker and therapeutic target in HCC.

## RESULTS

### EGR1 activates miR-203a expression

**Figure 1:**
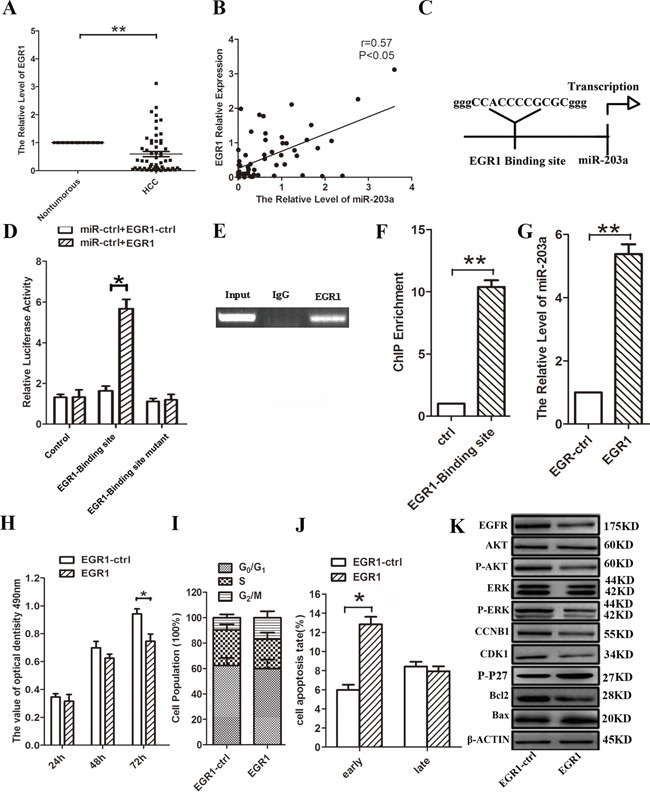
EGR1 induces miR-203a promoter activity in HCCs **A.** The expression levels of EGR1 mRNA in HCC and healthy tissues were analyzed by qRT-PCR. **B.** The relationship between EGR1 and miR-203a were assayed by Pearson's r. **C.** Schematic diagram of the putative miR-203a promoter with one potential EGR1 response element. **D.** Luciferase activity of reporter constructs spanning the putative EGR1 binding site or a negative control sequence. **E.** The interaction of EGR1 with miR-203a was shown using ChIP assays with control (rat IgG) or anti-EGR1 antibody. **F.** qRT-PCR analysis was performed with primers spanning predicted EGR1 of miR-203a. **G.** The expression of miR-203a was analyzed by qRT-PCR after transfection with EGR1 vector or empty vector in SMMC-7721 cells. **H-J.** MTT assay, cell cycle and cell apoptosis were performed to determine the impact of HCCs treated with EGR1 expression vector. **K.** Proposed model for HOXD3-mediated effects of miR-203a on the EGFR-AKT/MAPK pathways. The expression of EGFR, AKT, p-AKT, ERK, p-ERK, CCNB1, CDK1, p-P27, Bcl-2 and Bax was detected by western blot (*: p < 0.05, **: p < 0.01.).

Using quantitative real-time PCR (qRT-PCR), we found lower expression of EGR1 mRNA levels in HCC tissues, compared with their respective healthy tissues (Figure 1A). The levels of miR-203a were correlated with EGR1 expression (Figure 1B). To elucidate the mechanism underlying the downregulation of miR-203a in HCC, we used the UCSC genome browser tool and identified putative binding sequences for EGR1 located 0.6 kb upstream of the miR-203a locus (Figure 1C). Transfection of HCC cells with a luciferase construct including a 300-bp DNA fragment covering the putative EGR1 binding site upstream of the luciferase reporter resulted in increased luciferase expression compared to controls. However, luciferase activity was not changed when the construct included a mutated putative EGR1 binding sequence (Figure 1D). Consistent with these results, ChIP analysis revealed the EGR1 protein bound to the putative binding site upstream of miR-203a (Figure 1E and 1F). Overexpression of EGR1 in HCC cells resulted in increased miR-203a expression, inhibition of cell proliferation, and enhanced apoptosis (Figure 1G-1J). Furthermore, overexpression of EGR1 decreased the expression of cell cycle and apoptosis-related proteins, EGFR, p-AKT, p-ERK, CCNB1, CDK1 and Bcl2, suggesting that EGR1 contributes to the modulation of miR-203a transcription (Figure 1K and Figure S1A). This in turn suggests that miR-203a could be directly activated by the EGR1 transcription factor in HCC cells. The correlation between EGR1 expression levels and clinicopathological characteristics of HCC patients is summarized in Table 1. Strikingly, low EGR1 levels were significantly associated with poor tumor histology (well: 68.4% (13/19); moderate: 55.6% (5/9); poor: 90.0% (27/30)) (P = 0.040), but not with age, gender, or TNM stage. This suggests that EGR1 might be involved in the progression of HCC.

**Table 1:** Patient characteristics and clinicopathologic correlation of HOXD3 and EGR1 expression

Characteristics	Number of cases	HOXD3 expression		P-value	EGR1 expression		P-value
		High	Low		High	Low	
Age (years)				0.983			0.358
≥60	25	19	6		4	21	
<60	33	25	8		9	24	
Gender				0.291			0.534
Male	44	35	9		9	35	
Female	14	9	5		4	10	
Histology				0.023			0.040
Well	19	11	8		6	13	
Moderate	9	6	3		4	5	
poor	30	27	3		3	27	
pTNM Stage				0.048			0.799
I	11	9	2		2	9	
II	14	7	7		4	10	
III	27	23	4		5	22	
IV	6	5	1		2	4	

### MiR-203a expression levels affect SMMC-7721/Hep3B cell progression *in vitro*

To validate the role of miR-203a in liver cancer, we analyzed the expression of miR-203a in 58 pairs of HCC and normal liver tissues by real-time PCR. Compared to expression in normal tissues, miR-203a is significantly downregulated in 72% (42/58) of hepatocellular carcinoma samples (Figure S2A). In addition, analysis of miR-203a expression in 5 HCC cell lines (Hep3B, SMMC-7721, HepG2, Bel7402, and Huh7) revealed that miR-203a is downregulated in tumor cells as well (Figure S2B). These data indicate that miR-203a may act as a tumor suppressor in HCC.

**Figure 2:**
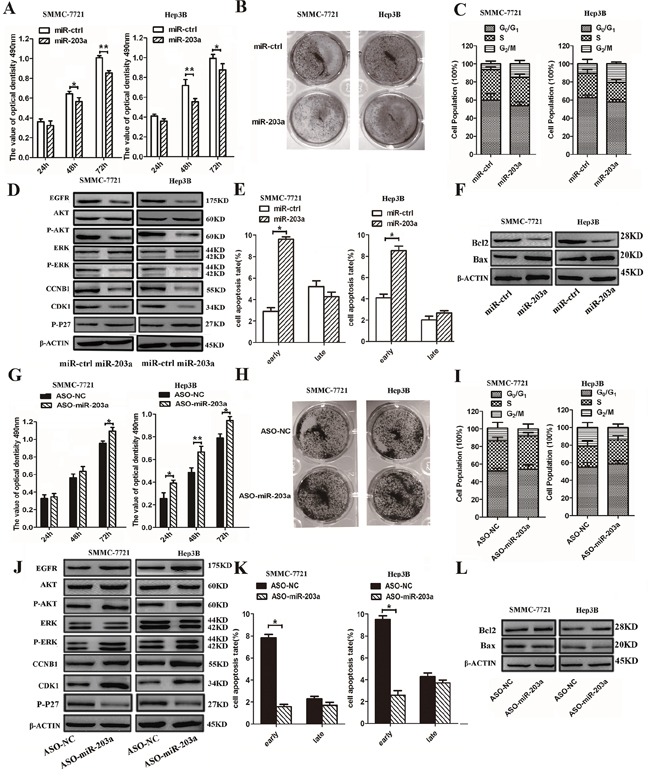
MiR-203a inhibits HCC cell proliferation and promotes apoptosis *in vitro* **A.** SMMC-7721/Hep3B cells were transfected with miR-203a vector or control vector and the effects were determined by MTT assay after 24, 48, and 72 h. **B.** Representative results of a colony formation assay for SMMC-7721/Hep3B cells after miR-203a overexpression. **C.** SMMC-7721/Hep3B cells were transfected with miR-203a vector and control vector. After 24 h, cell cycle distribution was analyzed by flow cytometry. A histogram indicates the percentage of cells in G0/G1, S, and G2/M cell cycle phases. **D.** The expression of EGFR, AKT, p-AKT, ERK, p-ERK, p-P27, CCNB1 and CDK1 was analyzed by western blot. **E.** Apoptosis was detected by annexin-V/propidium iodide combined labeling flow cytometry in SMMC-7721/Hep3B cells 24 h after transfection with miR-203a or control vector. Apoptotic evaluation was carried out by calculating the percentage of apoptotic cells. **F.** Analysis of the expression of apoptosis-related proteins in SMMC-7721/Hep3B cells after transfection with miR-ctrl or a miR-203a overexpression construct. **G.** MTT assay of SMMC- 7721/Hep3B cells after miR-203a overexpression. **H.** The growth of SMMC-7721/Hep3B cells was detected by colony formation. **I.** Cell cycle was determined in SMMC-7721/Hep3B cells transfected with ASO-miR-203a or a negative control. **J.** The expression of EGFR, AKT, p-AKT, ERK, p-ERK, CCNB1 and CDK1 was analyzed by western blot. **K.** Apoptosis was determined in SMMC-7721/Hep3B cells transfected with ASO-miR-203a or a negative control. **L.** the expression of Bcl-2 and Bax were detected by western blot (*: P < 0.05, **: P < 0.01).

The results of qRT-PCR showed that expression of miR-203a was increased by more than 30- and 20-fold in miR-203a-transfected SMMC-7721 and Hep3B cells, respectively, when compared with cells transfected with the control vector (miR-ctrl) (Figure S2C). To examine the role of miR-203a in HCC growth, we employed MTT, colony formation, and cell cycle analysis assays. The assay results showed that transient overexpression of miR-203a led to inhibition of proliferation of SMMC-7721/Hep3B cells at 48 and 72 h post-transfection (Figure 2A). Over-expression miR-203a also reduced colony formation in comparison with the miR-ctrl (Figure 2B). To further investigate the mechanisms by which miR-203a inhibit HCC cell proliferation, we adopted a serum starvation-stimulation strategy to determine whether miR-203a-induced inhibition of proliferation resulted from cell cycle checkpoint blockage. Overexpression of miR-203a resulted in a marked accumulation of HCC cells in G2/M phase (Figure 2C), suggesting that miR-203a indeed blocked transition out of the G2/M phase. In addition, the expression levels of the cell cycle regulators EGFR, p-AKT, p-ERK, CCNB1, and CDK1 were analyzed via western blot and found to be downregulated, whereas the expression of p-P27 was upregulated (Figure 2D and Figure S1B). Overexpression of miR-203a in SMMC-7721/Hep3B cells also led to an increase in apoptosis, compared to cells transfected with a control vector (Figure 2E). In addition, overexpression of mir-203a resulted in increased Bax expression and inactivation of Bcl-2 (Figure 2F). These data demonstrate that miR-203a induces apoptosis in HCC cells *in vitro*.

Furthermore, to examine the anti-proliferative role of miR-203a in human HCC cells, we depleted endogenous miR-203a in SMMC-7721/Hep3B cells by applying its inhibitor, ASO-miR-203a. Reduced endogenous expression of miR-203a in SMMC-7721/Hep3B cells increased cell viability and colony formation potential (Figure 2G and 2H). Further investigation into the effects of ASO-miR-203a on cell cycle progression in SMMC-7721/Hep3B cells demonstrated that G2/M translation was increased with ASO-miR-203a transfection (Figure 2I). We also studied the expression of cell-cycle-regulating proteins during overexpression of mir-203a in HCCs (Figure 2J and Figure S1B). Using a cellular apoptosis assay, we found the opposite result in SMMC-7721/Hep3B cells transfected with the miR-203a inhibitor as we did with the miR-203a overexpression. The apoptotic rate was decreased in ASO-miR-203a transfected cells compared to cells transfected with a control plasmid, and although the expression of Bax was decreased, Bcl-2 expression was increased (Figure 2K and 2L). These results suggest that endogenous miR-203a plays an essential anti-carcinogenic role in HCCs preventing liver cancer progression.

### HOXD3 is a direct target of miR-203a

**Figure 3:**
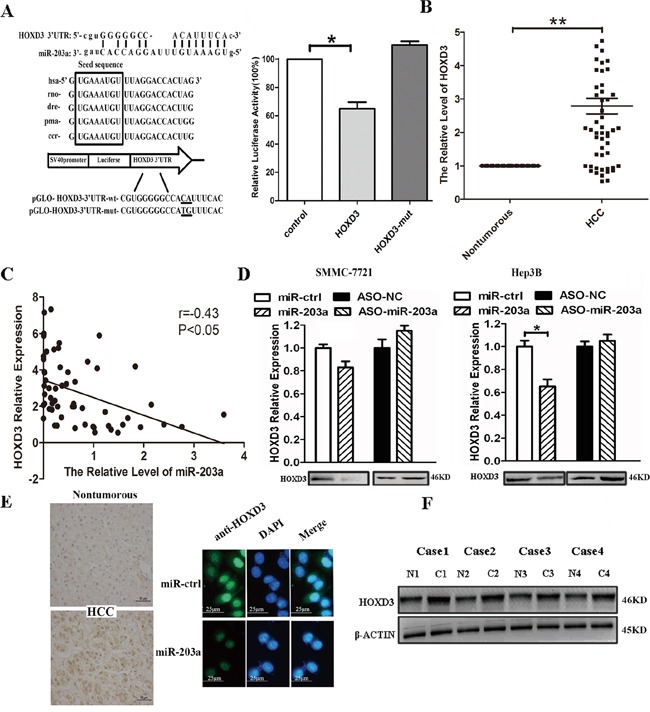
MiR-203a inhibits tumor proliferation by directly targeting HOXD3 in hepatocellular carcinoma **A.** miR-203a is highly conserved across species and it has binding sites within the 3′-UTR of human HOXD3. A luciferase assay was performed in SMMC-7721 cells in which miR-203a was co-transfected with pGLO-HOXD3 or pGLO-HOXD3 mutant vector. **B.** mRNA expression levels of HOXD3 were measured by RT-PCR in tumor tissues and normal tissues. **C.** There is an inverse correlation between miR-203a and HOXD3 expression in HCC tissues (r = −0.38, *: P < 0.05). **D.** mRNA and protein expression levels of HOXD3 were measured by RT-PCR and western blot in SMMC-7721/Hep3B cells transfected with miR-203a, ASO-miR-203a, or a negative control. **E.** HOXD3 protein expression levels were measured by IHC and ICC in HCC tissues and SMMC-7721 transfected with miR-203a. **F.** HOXD3 protein levels were measured by western blotting for 4 paired HCC and healthy tissue samples (*: P < 0.05, **: P < 0.01).

To experimentally confirm whether miR-203a directly targets HOXD3 in HCC cells, we constructed 3′-UTR fragments of HOXD3 containing the potential binding site for miR-203a. Both wild-type (HOXD3-WT) and mutant (HOXD3-MT) binding sites were subcloned into the region downstream of the pmiRGLO dual-luciferase reporter vector. HCC cells were co-transfected with miR-203a and the WT or MT 3′-UTR vector. We observed a remarkable reduction in luciferase activity from the HOXD3-WT reporter constructs in HCC cells transfected with miR-203a, but not from the reporter vector containing a mutant binding site. These findings indicate that miR-203a could bind directly to the HOXD3 3′-UTR (Figure 3A).

Next, we used RT-PCR to investigate the relationship between miR-203a and HOXD3 at the mRNA level. We found the HOXD3 mRNA expression was higher in HCC tissues than in healthy paired liver tissues (Figure 3B). Likewise, miR-203a levels were inversely correlated with HOXD3 expression (Figure 3C). Next, we verified the expression of HOXD3 at both the mRNA and protein level in SMMC-7721/Hep3B cells transfected with miR-203a or miR-203a inhibitor. The expression of the HOXD3 protein was decreased in SMMC-7721/Hep3B cells, whereas knockdown of miR-203a enhanced HOXD3 protein expression (Figure 3D). In addition, immunohistochemistry analysis showed that the expression of HOXD3 was increased in HCC tissues compared to healthy tissues. Immunofluorescence microscopy was used to evaluate the expression of HOXD3 in SMMC-7721 cells transfected with miR-203a or its negative control (Figure 3E). Four pairs of HCC tissues were chosen to analyze HOXD3 protein expression via western blot. HOXD3 expression was higher in cancer tissue than in normal tissue (Figure 3F). Correlations between HOXD3 expression levels and the clinicopathological characteristics of HCC patients are summarized in Table 1. Strikingly, high HOXD3 levels were significantly associated with poor tumor histology (well: 57.9% (11/19); moderate: 66.7% (6/9); poor: 90.0% (27/30) (P = 0.023), but not with age or gender, suggesting that upregulated HOXD3 protein expression might be involved in the progression of HCC. Overall, the above data suggests that miR-203a is able to directly regulate HOXD3 expression in HCC cells.

### Knockdown of HOXD3 reduces the progression of HCC cells and overexpression of HOXD3 eliminates the effects of miR-203a

**Figure 4:**
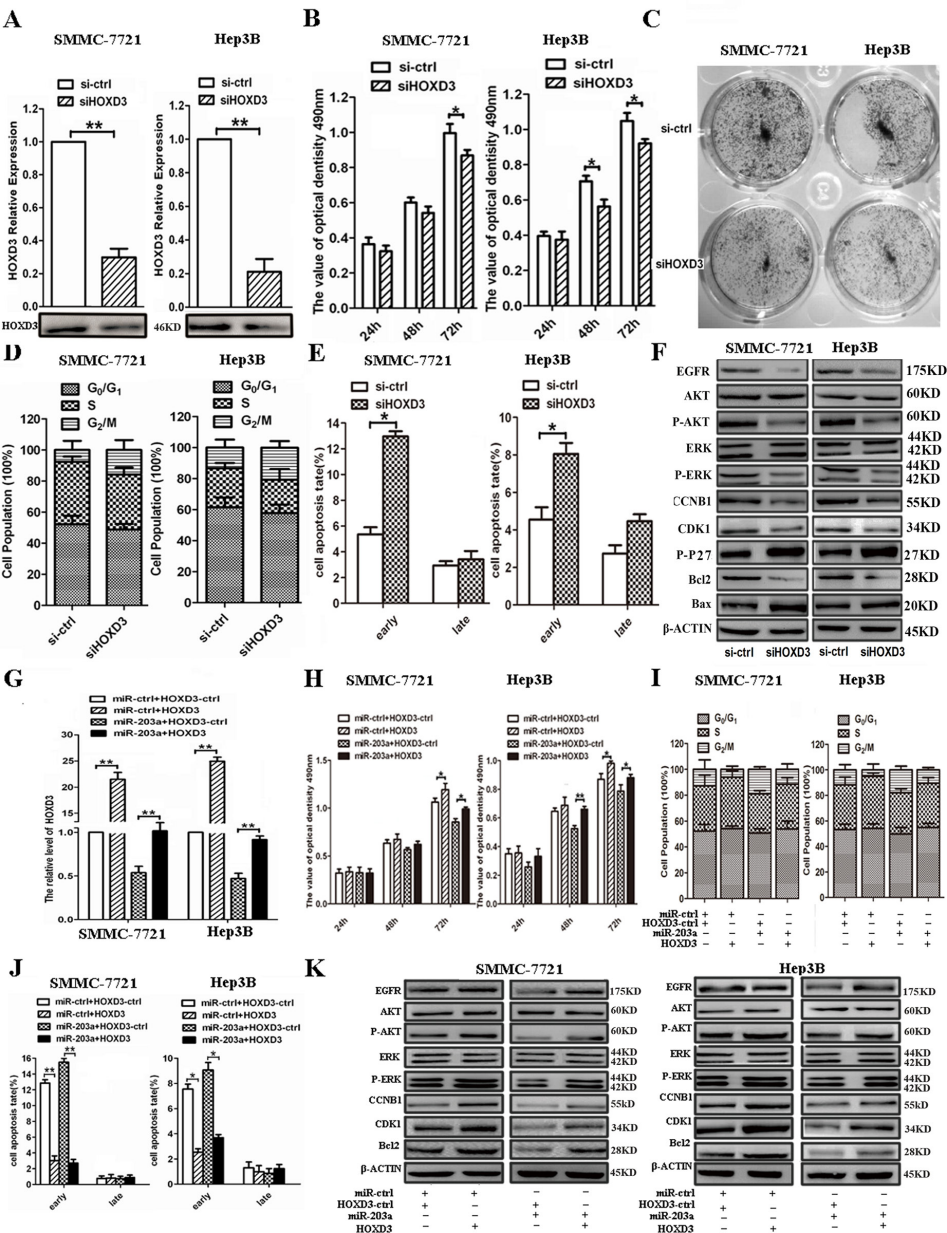
Expression levels of HOXD3 affect HCC progression **A.** The expression levels of HOXD3 were measured by qRT–PCR (upper panel) and western blot (lower panel) in SMMC-7721/Hep3B cells transfected with siHOXD3. **B.** An MTT assay was performed to determine the growth of HCCs treated with siHOXD3 or a negative control (si-ctrl). Data are reported as mean ± s.d. for 3 independent experiments. **C.** The colony formation assay was performed 7 days after transfection of HCCs with siHOXD3 or a negative control (si-ctrl). **D.** Cell cycle progression was determined in HCCs 48 h after transfection with siHOXD3 by propidium-iodide staining and flow cytometry. The histogram indicates the percentage of cells in G0/G1, S, and G2/M cell-cycle phases. **E.** Apoptosis was determined in HCCs 48 h after transfection with siHOXD3. **F.** Protein expression of HOXD3-dependent cell cycle or apoptosis-related proteins in HCCs transfected with siHOXD3 or si-ctrl-HCCs was analyzed by western blot. **G-K.** qRT-PCR, MTT assay, cell cycle, cell apoptosis and western blot were performed to determine the impact of HCCs treated with HOXD3 expression vector (*: P < 0.05, **: P < 0.01).

Next, we silenced HOXD3 expression by RNAi to test whether HOXD3 is involved in the anti-tumor effects of miR-203a. HOXD3-specific knockdown can be achieved using targeted siRNA in SMMC-7721/Hep3B cells (Figure 4A). Silencing HOXD3 resulted in suppressed cell proliferation, G2/M arrest, and increased cell apoptosis, similar to the effects of miR-203a overexpression in SMMC-7721/Hep3B cells (Figure 4B-4E). Based on western blot analysis, HOXD3 siRNA reduced the expression of not only HOXD3 but also EGFR, p-AKT, p-ERK, CCNB1, CDK1, and Bcl2 and it promoted the expression of p-P27 and Bax (Figure 4F and Figure S1C). Based on these findings, we concluded that miR-203a can regulate HCC progression by directly targeting HOXD3.

To further demonstrate that miR-203a suppressed tumor progression through targeting HOXD3, we constructed a HOXD3 overexpression vector and co-transfected it with miR-ctrl or miR-203a into SMMC-7721/Hep3B cells. Overexpression of HOXD3 in SMMC-7721/Hep3B cells was able to rescue the cells from the silencing effects of miR-203a on HOXD3 expression (Figure 4G). In addition, we found that HOXD3 overexpression counterbalanced the tumor suppressing effect of miR-203a on HCC cell proliferation (Figure 4H). We also discovered that overexpression of HOXD3 increased G2/M translation and decreased cell apoptosis in SMMC-7721/Hep3B cells (Figure 4I and 4J). Co-transfection with miR-203a and HOXD3 also led to activation of G2/M translation and inactivation of apoptosis in HCC cells. Furthermore, the expression of EGFR, p-AKT, p-ERK, CCNB1, CDK1, and Bcl2 was upregulated after transfection with HOXD3, but the expression levels of these regulators were unaltered after co-transfection with both miR-203a and HOXD3 constructs (Figure 4K and Figure S1D). These results further suggest that miR-203a exhibits its tumor suppressor role by directly targeting HOXD3.

### MiR-203a induces growth inhibition of SMMC-7721 cells *in vivo*

**Figure 5:**
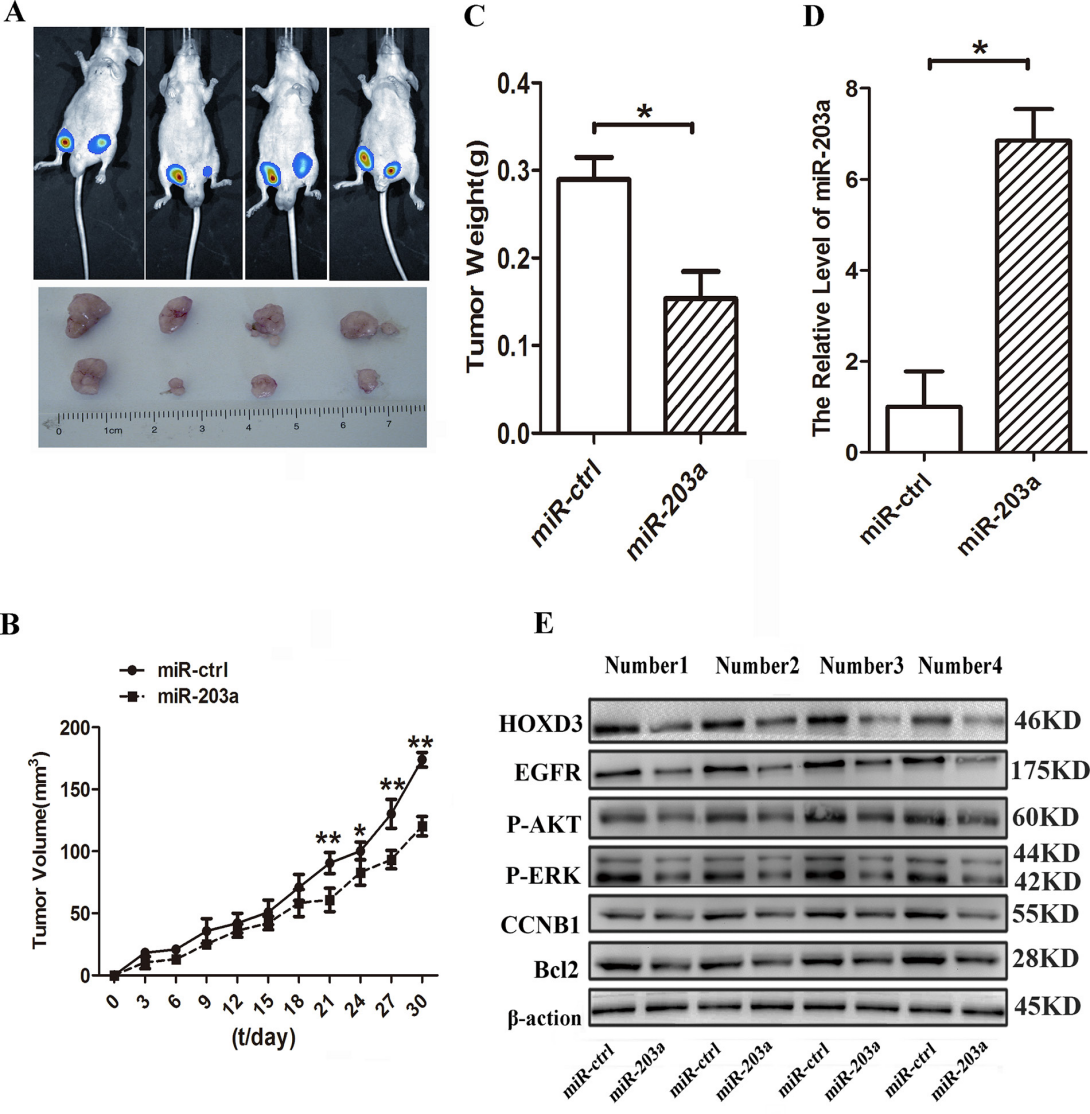
MiR-203a inhibits hepatocellular carcinoma progression *in vivo* **A.** Tumor growth was analyzed through imaging of small animals at day 25. **B.** Tumor growth curves. **C.** Tumor weight. **D.** The expression levels of miR-203a were analyzed by qRT-PCR analysis in the tumor tissues from the animal. **E.** The expression levels of HOXD3, EGFR, p-AKT, p-ERK, CCNB1, and Bcl2 were analyzed by western blot in tissues from the animal (*: P < 0.05, **: P < 0.01, Student's *t*-test).

To further confirm the inhibitory function of miR-203a in HCC, we used lentiviral vectors to stably restore the expression of miR-203a in SMMC-7721 cells. Cells infected with either a lentiviral vector expressing mir-203a (LV-miR-203a) or a control (LV-CN) sequence were injected subcutaneously into opposite posterior flanks of the same nude mice. We measured xenograft tumor growth for 4 weeks. As shown in Figure 5A, tumor growth was significantly suppressed by LV-miR-203a compared with the control, at least for the duration of the experiment. On day 30, the average volume of miR-203a-treated tumors was much smaller than that of control tumors (Figure 5B). Average tumor weights for control and miR-203a groups on day 30 were 0.27 g and 0.16 g, respectively (Figure 5C). Furthermore, expression levels of miR-203a and HOXD3 in tumor tissues were examined by qRT-PCR and western blot. Consistent with the *in vitro* data, our *in vivo* data showed that expression of miR-203a was increased and expression of the HOXD3 was reduced in miR-203a-treated tumors, whereas the expression of EGFR, p-AKT, p-ERK, CCNB1, and Bcl2 was downregulated (Figure 5D and 5E). These data indicate that miR-203a expression is capable of inhibiting tumor growth and HOXD3 expression *in vivo*.

### EGR1 mediates miR-203a suppress the HCCs progression by targeting HOXD3 through EGFR signaling pathway

**Figure 6:**
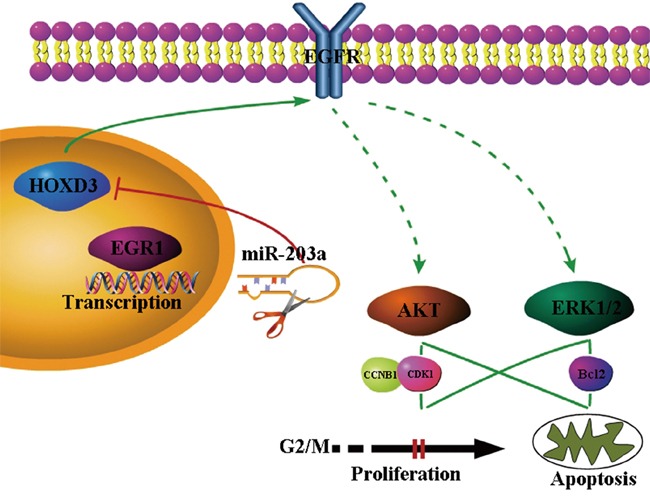
Proposed model for MiR-203a was directly activated by the EGR1 transcription factor Expression of miR-203a downregulated the target genes of HOXD3. HOXD3 is essential for the activation of direct EGFR and indirect (p-ERK1/2 and p-AKT through PI3K or MAPK) signaling pathways in HCC cells.

EGR1 bind to the miR-203a promoter sequence and active the expression of miR-203a. Activated miR-203a could suppress the hepatocellular carcinoma cells progression and induce the cell apoptosis by targeting HOXD3 through EGFR/AKT and ERK signaling pathway (Figure 6).

## DISCUSSION

In agreement with the role of EGR1 in non-small cell lung cancers, breast cancer, glioblastomas, and acute myelogenous leukemia [6, 7, 28], our data support the hypothesis that EGR1 acts as a tumor suppressor in HCC. In our research, the expression of EGR1 was assayed by qRT-PCR, and found to be downregulated in HCC tissues, compared to in healthy tissues. This result coincided with the expression pattern of EGR1 using the assay of Oncomine Platform and TCGA database (Figure S3A and S3B). Inaddition, we found that miR-203a levels were correlated with EGR1 expression levels, and that EGR1 directly binds to the miR-203a promoter, based on ChIP and dual luciferase assays. To the best of our knowledge, this is the first time that a relationship between EGR1 and miR-203a has been demonstrated.

Accumulating evidence shows that alterations in miRNA levels are involved in HCC and correlated with its biopathological and clinical features [29–31]. Using the TCGA database, we found that miR-203 is downregulated in HCC tissues (Figure S4), which is coincident with our results in this study (Figure S2). Although the role of miR-203a as a tumor suppressor in human renal cell carcinoma had been identified [32], the underlying mechanisms responsible for decreased expression of miR-203a in HCC are still unknown. Based on gain- and loss-of-function assays, our study shows that miR-203a decreases cell proliferation and clonogenicity, increases apoptosis, induces G2/M arrest, and decreases the expression of cell cycle/apoptosis-related proteins (including EGFR, p-AKT, p-ERK, CCNB1, CDK1 and Bcl2) both *in vitro* and *in vivo*. Therefore, our data provide a more comprehensive understanding of the tumor suppressor role of miR-203a during HCC development.

HOX represents a cluster of homeodomain genes encoding critical master regulatory transcription factors that determine cellular identity during development. So far, 39 HOX family genes have been identified [33], but their function in the regulation of cellular progression remains largely unknown. Recently, studies have demonstrated that HOX family genes play key roles in tumorigenesis. Several studies on cluster D genes of the HOX family have been published. High expression of HOXD9 was reported in SK-MG1 cells and in human glioma cancer stem cells [34]. Another study reported that downregulation of HOXD10 expression by miRNA-10b leads to an increase in pro-metastatic gene products [35]. HOXD3 mRNA expression levels in hormone receptor-negative breast cancer are significantly higher than in non-cancerous tissues, whereas in colon carcinoma they are downregulated [36]. In this study, we used the TCGA database to test the expression of HOXD3 in liver cancer and normal tissues, and found that the expression of HOXD3 was increased in liver cancer tissues compared with their normal counterparts (Figure S5A), which was consistent with our results. MiRNAs cause mRNA degradation or inhibition of mRNA translation by interacting with the 3′-UTR of target gene mRNA [37]. Using a dual-luciferase reporter assay, we demonstrated that miR-203a directly targets HOXD3 by recognizing its 3′-UTR and inhibiting translation. In a study on breast cancer, Shaoqiang et al. found that high levels of HOXD3 expression were associated with significantly shorter survival rates [38]. Interestingly, our study also revealed that high levels of HOXD3 expression were related to histological findings in HCC. In addition, via HOXD3 gain- and loss-of-function studies, we discovered that overexpression of HOXD3 activates genes involved in MAPK/AKT cell signaling pathways, inducing cell proliferation and suppressing apoptosis in HCCs.

In addition, we demonstrated that aberrant expression of HOXD3 affects the expression of EGFR. Using the UCSC genome browser VISTA tool (http://genome.lbl.gov/vista/index.shtml) and luciferase assays, we found that HOXD3 could target the upstream region of EGFR (Figure S5B and S5C). This suggests that HOXD3 might be involved in regulation of EGFR in HCC. Next, we found that EGFR expression is elevated in liver cancer tissues in comparison to normal tissues using TCGA database and expression of EGFR is correlated to expression of miR-203a (Figure S5D-S5F). Cell cycle control and apoptosis are the major regulatory mechanisms for cell growth and death. Many genes and cell signaling pathways are involved in these two biological processes, including the MAPK and PI3K/AKT pathways [39, 40]. The cell cycle is coordinated by a number of proteins including cyclins and cyclin-dependent kinases (CDKs), which exist as inactive serine/threonine kinase monomers that become activated when bound to specific cyclins. Cyclins are nuclear proteins that are transiently expressed in order to activate their corresponding CDKs, and their activities are inhibited by two families of CDK inhibitors (CKIs) [41]. During the transition from G2 to M phase, cyclinB1, CDK1, as well as the cyclinB1/CDK1 complex are recruited to regulate the progression of cells [42]. Here, we observed that the expression of p-AKT, p-ERK1/2, CDK1, and CCNB1 was downregulated by miR-203a expression, resulting in G2/M arrest in SMMC-7721 and Hep3B cells. We first demonstrated that miR-203a could suppress cell proliferation via HOXD3/MAPK and HOXD3/AKT cell signaling pathways. Furthermore, previous research shows that ERK1/2 and AKT can initiate the mitochondria-dependent intrinsic cell death pathway by direct phosphorylation of Bax, a member of the Bcl-2 family of apoptotic proteins [43, 44]. In our current study, we verified that downregulation of ERK1/2 and AKT induced a decrease in the expression of Bcl-2, and an increase in the expression of Bax, when miR-203a was overexpressed.

In summary, we provide novel evidence that transcription factor EGR1 can directly increase the expression of miR-203a and indirectly downregulate the expression of HOXD3. In addition, we show that miR-203a is able to suppress cell growth and increase apoptosis *in vitro* and *in vivo* by targeting HOXD3. HOXD3 can bind to the EGFR promoter sequence, leading to inhibition of cell proliferation and promotion of apoptosis through the EGFR-MAPK/AKT pathway. Thus, it may be a potential therapeutic strategy for HCC in the future.

## MATERIALS AND METHODS

### Cell lines and tissue samples

BEL7402, SMMC-7721, HepG2, Hep3B, Huh7 and HL-7702 cells were cultured in 1640 medium (PAA Laboratories GmbH, Pasching, Austria), supplemented with 10% fetal bovine serum (PAA Laboratories GmbH, Pasching, Austria) at 37°C in a humidified chamber with 95% air and 5% CO2. A total of 58 paired HCCs and adjacent non-tumor liver tissue samples were collected from patients undergoing resection of HCC at the Hepatobiliary Surgery and Pathology Department of the First and Second Affiliated Hospital of Xi'an Jiaotong University, China. None of the patients received chemotherapy or radiotherapy before surgery. Tissue samples were immediately snap frozen in liquid nitrogen until RNA extraction. Both tumor and non-tumor tissues were histologically confirmed. Informed consent was obtained from each patient and the study was approved by the Institute Research Ethics Committee of The Cancer Center of Xi'an Jiaotong University.

### Plasmid

A 300 bp sequence comprising the EGR1 binding site and binding site mutant were cloned into the pGL3-promoter vector between the *Sac*I and *Xho*I sites. The miR-203a expression vector was constructed by amplifying miR-203a with synthetic oligonucleotides and cloning it in between the *Eco*RI and *Hin*dIII sites of the pcDNA6.2-GW/EmGFP vector (Invitrogen). The software program RegRNA (regulatory RNA motifs and elements finder; http://regrna.mbc.nctu.edu.tw/) was used to predict gene-related specified microRNA targets. Using bioinformatics analyses, we identified a fragment of HOXD3 as a miR-203a target. The 3′-UTR of human HOXD3 mRNA was constructed by synthetic oligonucleotides and cloned in between the *Sac*I and *Xho*I sites of the pmirGLO Dual-Luciferase miRNA Target Expression Vector (Promega). The inhibitor of miR-203a and small interfering RNA (siRNA) targeting HOXD3 were purchased from GenePharma. Sequence information on all the vectors is listed in Table 2.

**Table 2:** Primers and oligonucleotides used in this work

Name	Sequence(5′-3′)
Pri-miR-203a-S	5′-GTGTTGGGGACTCGCGCGCTGGGTCCAGTGGTTCTTAACAGT TCAACAGTTCTGTAGCGCAATTGTGAAATGTTTAGGACCACTAGAC CCGGCGGGCGCGGCGACAGCGA-3′
Pri-miR-203a-A	5′-TCGCTGTCGCCGCGCCCGCCGGGTCTAGTGGTCCTAAACATTTCACA ATTGCGCTACAGAACTGTTGAACTGTTAAGAACCACTGGACC CAGCGCGCGAGTCCCCAACA-3′
HOXD3 3′UTR-S	5′-CCGTGGGGGCCACATTTCACC-3′
HOXD3 3′UTR-A	5′-TCGAGGTGAAATGTGGCCCCCACGGAGCT-3′
HOXD3 3′UTR-MS	5′-CCGTGGGGGCCATGTTTCACC-3′
HOXD3 3′UTR-MA	5′-TCGAGGTGAAACATGGCCCCCACGGAGCT-3′
siRNA-ctrl-S	5′-UUCUCCGAACGUGUCACGUTT-3′
siRNA-ctrl-A	5′-ACGUGACACGUUCGGAGAATT-3′
siHOXD3-S	5′-GAGUCUCGACAGAACUCCATT-3′
siHOXD3-A	5′-UGGAGUUCUGUCGAGACUCTT-3′
miR-203a RT	5′-GTCGTATCCAGTGCGTGTCGTGGAGTCGGCAATTG CACTGGATACGACCTAGTGG-3′
miR-203a-F	5′-ATCCAGTGCGTGTCGTG-3′
miR-203a-R	5′-TGCTGTGAAATGTTTAGGA-3′
Inhibitor-ctrl	5′-CAGUACUUUUGUGUAGUACAA-3′
MiR-203a inhibitor	5′-CUAGUGGUCCUAAACAUUUCAC-3′
HOXD3-F	5′-TCAAGAAAACACACACATACATAATTG-3′
HOXD3-R	5′-TGCTGAATCCTGAGAGAGCTG-3′
EGR1-F	5′-AGCCCTACGAGCACCTGAC-3′
EGR1-R	5′-GGTTTGGCTGGGGTAACTG-3′
β-actin-F	5′-CGGGAAGCTTGTCATCAATGG-3′
β-actin-R	5′-GGCAGTGATGGCATGGACTG-3′
U6 RT	5′-GTCGTATCCAGTGCAGGGTCCGAGGTGCACTGGATACGACAAAATATGG-3′
U6-F	5′-TGCGGGTGCTCGCTTCGGCAGC-3′
U6-R	5′ CCAGTGCAGGGTCCGAGGT 3′

### Real-time PCR

Total RNA was isolated from cells and frozen tissues with TRIzol^®^ reagent (Invitrogen, Carlsbad, CA, USA) according to the manufacturer's protocol. For mRNA analyses, cDNA was generated using the PrimeScript^®^ RT reagent Kit following the manufacturer's instructions, while the mature miRNA was reverse transcribed using miRNA-specific primers for quantification of miR-203a. PCR amplification was performed using SYBR Premix Ex Taq II on an FTC-3000TM System (Funglyn Biotech Inc., Toronto, Canada). All primers are listed in Table 2. Each sample measurement was performed in triplicate and a dissociation curve analysis was plotted for each PCR. U6 and β-actin were used as controls for miRNA and mRNA levels, respectively. Relative quantification was done using the 2^−ΔΔCt^ method.

### Cell proliferation assay

SMMC-7721/Hep3B cells were plated in 96-well plates at a density of 3000 cells/well. At 24, 48 and 72 h after transfection, cell proliferation was analyzed using the (3-4, 5-dimethylthiazol-2-yl)-2, 5-diphenyl-tetrazolium bromide (MTT) assay. In this assay, 20 μL of MTT solution was added to the cells. After incubation for 4 h at 37°C, the supernatant was discarded and replaced with 150 μL dimethylsulfoxide (DMSO). Absorbance was measured in a microplate spectrophotometer. Each experiment contained three technical replicates and was repeated at least twice. The data were summarized as mean ± standard deviation (s.d.).

### Colony formation assay

Transfected SMMC-7721/Hep3B cells were re-suspended and seeded onto 12-well plates at a density of 2000 cells/well, incubated for two weeks, and then stained with 0.5% crystal violet for 30 min. Excess dye was rinsed off via two washes with PBS. Pictures were obtained using Quantity One^®^ software from Bio-Rad.

### Cell cycle analysis

At 24 h post transfection, HCC cells were harvested and fixed with 70% ethanol at 4°C overnight. Cells were centrifuged at 1,500 rpm for 5 min and incubated with 0.1 mg/mL RNase A and 0.05 mg/mL propidium iodide (PI) for 30 min at 4°C. Then, cells were analyzed by flow cytometry.

### Cell apoptosis analysis

Cell apoptosis was analyzed using an Annexin-V FITC Apoptosis Detection Kit (Invitrogen), according to the manufacturer's instructions. Cells were seeded in triplicate onto 12-well plates at a density of 1 × 10^6^ cells per well, transfected with DNA or siRNA, and after 24 h, they were analyzed using a flow cytometer (Becton). Apoptotic populations were determined using ModFit software.

### Western blot

Transfected HCC cells were lysed using RIPA buffer, supplemented with protease inhibitor (Invitrogen). The protein concentration was estimated using a quantitative analyzer (GeneQuant pro RNA/DNA). Proteins were separated via 8% to 10% SDS-PAGE (Invitrogen) and transferred to a nitrocellulose membrane, which was incubated with HOXD3, EGFR, EGR1, CCNB1, CDK1, p-AKT, AKT, ERK1/2, p-ERK1/2, Bax, P27, Bcl-2, or β-actin antibody (Cell Signaling Technology, diluted 1/1,000). The membranes were washed three times with TBST and incubated with a goat anti-rabbit antibody (Bioworld, diluted 1/5,000). Protein expression was normalized to the β-actin levels in each sample.

### Dual luciferase assay

HCC cells were seeded in a 96-well plate (Corning) at a density of 1 × 10^4^ cells per well, 1 day prior to transfection. The miR-203a expression vector was co-transfected with wild-type or mutated 3′-UTR HOXD3 reporter constructs and a blank pmirGLO Dual-Luciferase as a positive control using Lipofectamine 2000 according to the manufacturer's protocol (Invitrogen). In a second experiment, EGR1 expression vector (or empty vector) was co-transfected with wild-type or mutated EGR1 binding site reporter constructs and a blank pGL3-Luciferase as a positive control. Reporter gene assays were performed 24 h post transfection using the Dual Luciferase^®^ Reporter Assay System (Promega) according to the manufacturer's protocol. All experiments were performed at least 3 times.

### Immunohistochemistry

The tissue samples were sectioned at a thickness of 4 μm. Sections were deparaffinized with xylene and hydrated using graded alcohol, after which antigen retrieval and blocking were performed. Slides were incubated with primary antibody (HOXD3) at 4°C overnight, followed by incubation with secondary antibody. For signal detection, 3,3′-diaminobenzidine (DAB) and hematoxylin were used. The staining intensity was scored manually. A sample was considered to display high HOXD3 expression if the percentage of positive cells was > 50% in 5 random fields.

### Immunofluorescence microscopy

To determine the effect of miR-203a on the protein level of HOXD3, we also performed immunofluorescence staining using the HOXD3 antibody. After 48 hours, the transfected SMMC-7721 cell lines were fixed with 4%formaldehyde for 20 minutes, then incubated with 0.5% Triton X-100. Rabbit anti-HOXD3 antibody was used for immunofluorescence staining. After washed 3 times with PBS, the cells were incubated with a goat anti-rabbit antibody, and measured by immunofluorescence microscopy.

### Tumorigenicity assay in nude mice

Tumorigenicity was analyzed in 30-day-old female nude mice. HCC cells were transfected with LV-miR-203a (purchased from Shanghai Genechem Co., Ltd., China) and LV-CN and re-suspended in PBS. Then, 1 × 10^6^ cells were injected subcutaneously into both posterior flanks of the nude mice. Tumor size was measured using a vernier caliper every 3 days for 30 days. The mice were anaesthetized by intra-peritoneal injection of 1% pentobarbital sodium (50 mg/kg). The tumor was removed after induction of deep anesthesia and the incision was closed with surgical staples. Mice were euthanized 3 weeks after the injection. Tumor volumes (V) were calculated by measuring the length (L) and width (W) of tumors, using the formula: V = (L × W^2^) / 2. All animal experiments were approved by the Institutional Animal Care and Use Committee of Xi'an Jiaotong University.

### Chromatin immunoprecipitation assay (ChIP)

The binding of EGR1 to the promoter of miR-203a was tested using ChIP analysis. SMMC-7721 cells were cross-linked using 1% formaldehyde (BIO-RAD, USA) for 15 min at room temperature, and the reactions were quenched with glycine (0.125 M) during 30 min. The cells were rinsed twice with 5 mL PBS. After harvesting the cells, the nuclei were resuspended in Mg-NI, Mg-NI-XP40, Ca-NI (0.5 M EGTA addition) and lysis buffer (with Protease inhibitor). A sonicator was used to shear the cross-linked DNA to an average length of 100 to 500 bp and samples were centrifuged at 14,000 g to remove insoluble material. Of this sample, 100 μL was used as input for ChIP. HOXD3 was immunoprecipitated from the supernatant using anti-HOXD3 antibody (mouse IgG was used as negative control) at 4°C for 4 h, and bound to protein G Sepharose (Invitrogen, USA) during a 2-h incubation at 4°C. The immunoprecipitates were washed twice with 1 mL of each of the following solutions: ChIP lysis buffer, ChIP lysis buffer containing 500 mM NaCl, LiCl/detergent solution (10 mM Tris-HCl, pH 8.0, 250 mM LiCl, 0.5% NP-40, 0.5% sodium deoxycholate, and 1 mM EDTA), and finally TE buffer (10 mM Tris and 1 mM EDTA, pH 8.0). Bound proteins were eluted from the beads using 1% SDS and 0.1 M sodium bicarbonate solution. The input and the eluent samples were reverse-cross-linked with proteinase K by incubating at 65°C for 8 h. The DNA from the samples was isolated by phenol/chloroform (Invitrogen, USA), followed by ethanol precipitation. Promoter binding was tested by PCR using primers spanning the upstream regions of the miR-203a start sites.

### Statistical analysis

All data are presented as mean ± SEM of at least 3 independent experiments. Student's *t*-test was used to compare 2 independent groups. The relationships between the expression of miR-203a, HOXD3, and EGR1, and the clinical and pathological characteristics were analyzed with the Chi-square test. Pearson's r was used to explore the association between miR-203a, and HOXD3 and EGR1 expression. All differences were considered statistically significant at P < 0.05.

## SUPPLEMENTARY FIGURES


