# Supplementary material for: EGR1 mediates miR-203a suppress the hepatocellular carcinoma cells progression by targeting HOXD3 through EGFR signaling pathway
Source: Oncotarget. 2016 May 26;7(29):45302–16. doi: 10.18632/oncotarget.9605 (PMC5216724; doi:10.18632/oncotarget.9605)
Supplement: Supplementary file 1 [file oncotarget-07-45302-s001.pdf]

EGR1 mediates miR-203a suppress the hepatocellular carcinoma cells progression by targeting HOXD3 through EGFR signaling pathway

SUPPLEMENTARY FIGURES

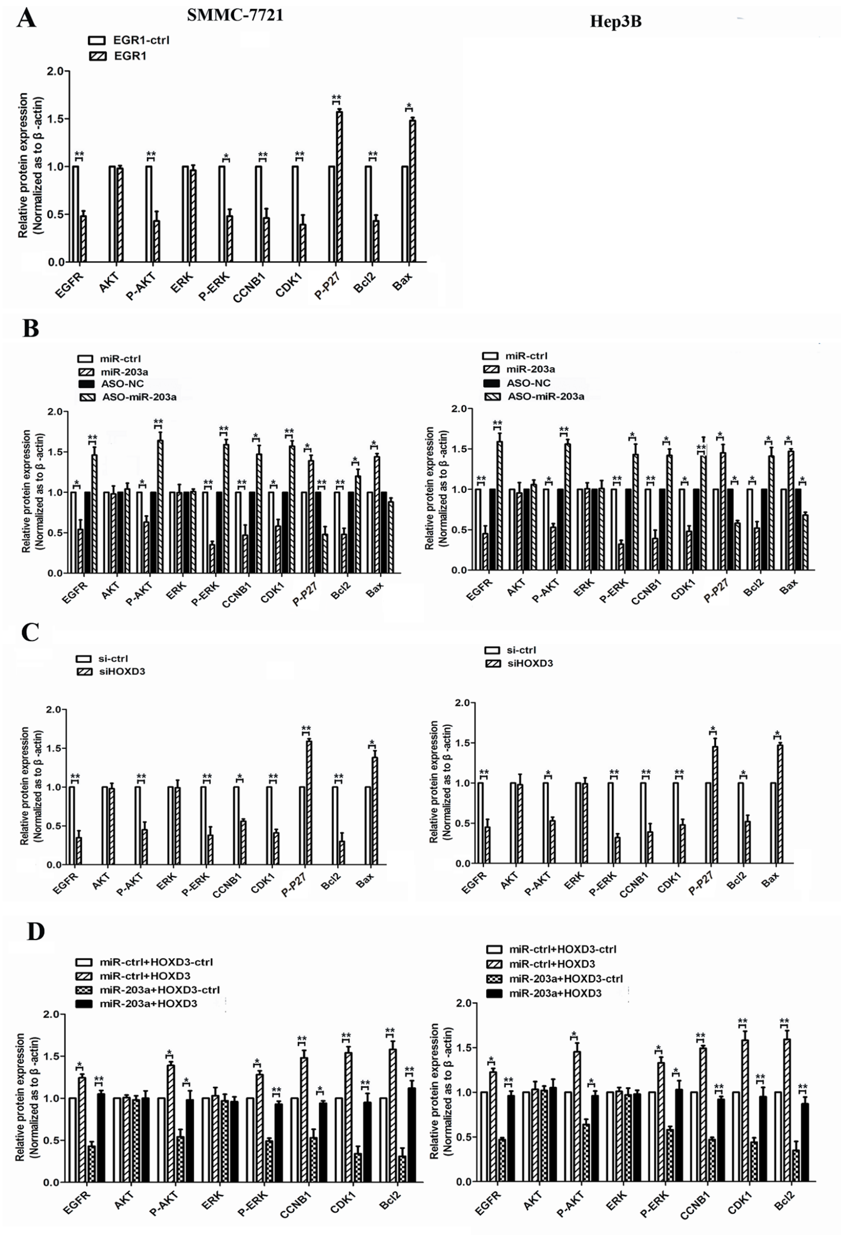

Supplementary Figure S1: The expression of cell cycle and apoptosis-related regulators in HCCs. A. The expression of EGFR, AKT, p-AKT, ERK, p-ERK, CCNB1, CDK1, p-P27, Bcl-2 and Bax was detected by western blot after transfected with EGR1-ctrl and EGR1, with β-actin as an internal control. The intensity for each band was quantified. **B.** Analysis of the expression of cell cycle and apoptosis-related regulators in SMMC-7721cells and Hep3B after transfected with miR-ctrl, miR-203a overexpression construct, ASO-NC, ASO-miR-203a. β-actin as an internal control. The intensity for each band was quantified. **C.** Analysis of the expression of cell cycle and apoptosis-related regulators in SMMC-7721cells and Hep3B after transfected with si-ctrl and siHOXD3 vector. The intensity for each band was quantified (*: $p < 0.05$, **: $p < 0.01$.). **D.** The expression of cell cycle and apoptosis-related regulators was detected by western blot after transfected with miR-ctrl and HOXD3-ctrl, miR-ctrl and HOXD3, miR-203a and HOXD3-ctrl, miR-203a and HOXD3, with β-actin as an internal control. The intensity for each band was quantified.

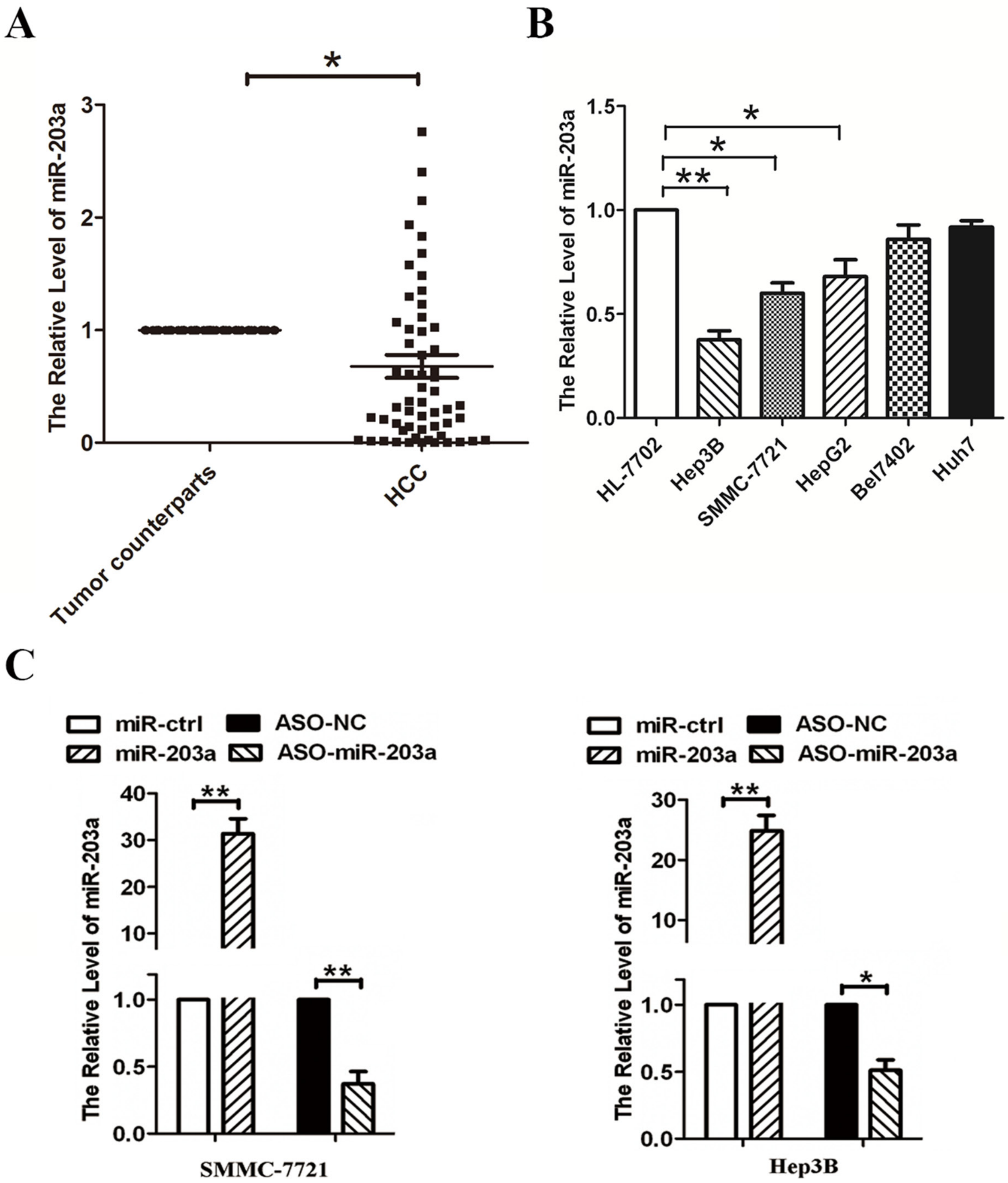

Supplementary Figure S2: Dysregulated miR-203a in hepatocarcinoma tissues and HCCs. A–B. qRT-PCR was performed to examine miR-203a expression in 58 paired human HCC tissues and HCC cells, adjacent nontumor tissues and HL-7702 cells as their respective control. **C.** qRT-PCR analysis of miR-203a in SMMC-7721/Hep3B cells transfected with miR-ctrl, miR-203a overexpression construct, ASO-NC, ASO-miR-203a (*: P < 0.05, **: P < 0.01).

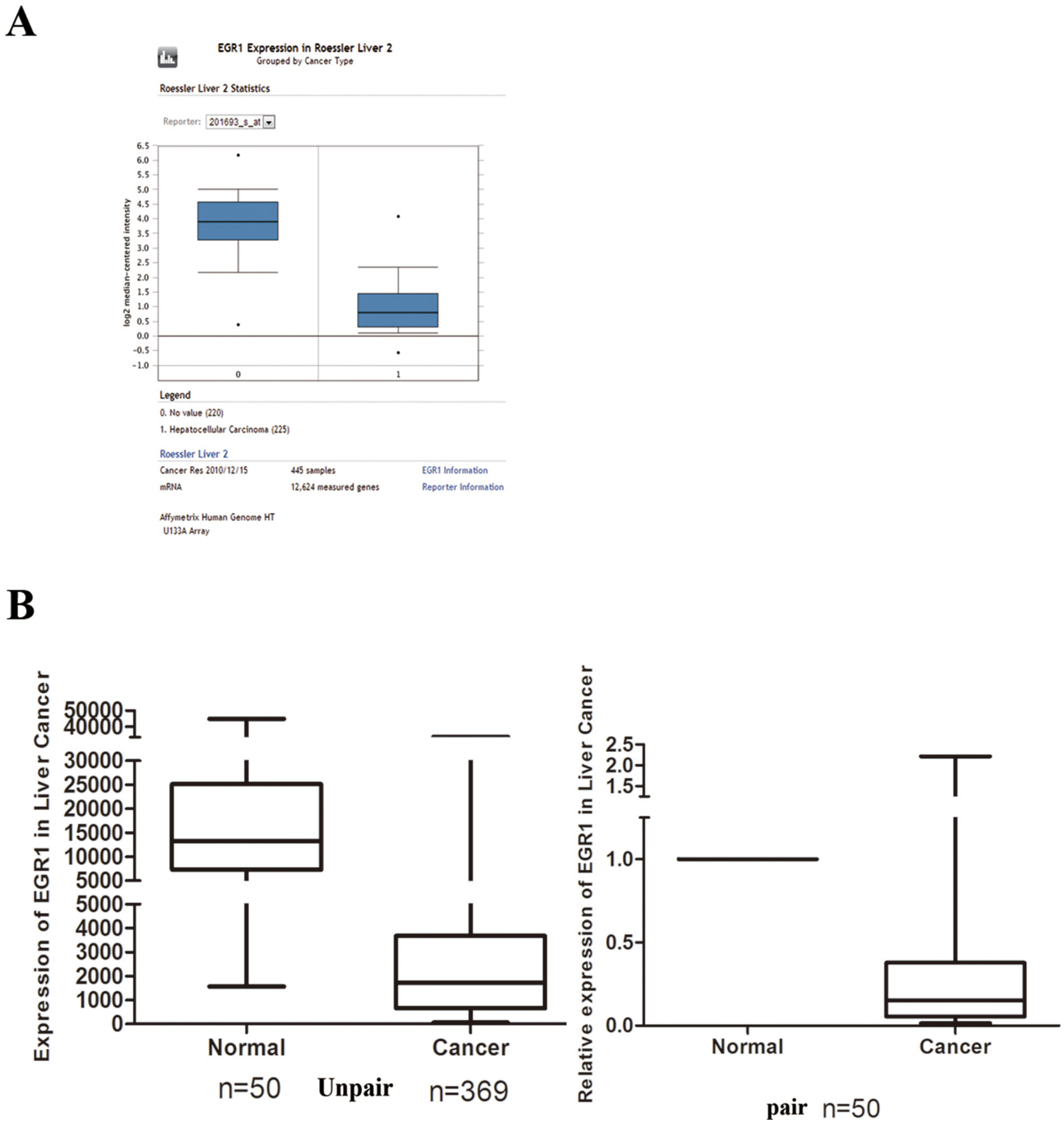

Supplementary Figure S3: The expression of EGR1 in hepatocarcinoma tissues. A. The expression of EGR1 in hepatocarcinoma tissues using the assay of Oncomine Platform database. **B.** The expression of EGR1 in paired or unpaired HCC tissues using the assay of TCGA database (*: $P < 0.05$).

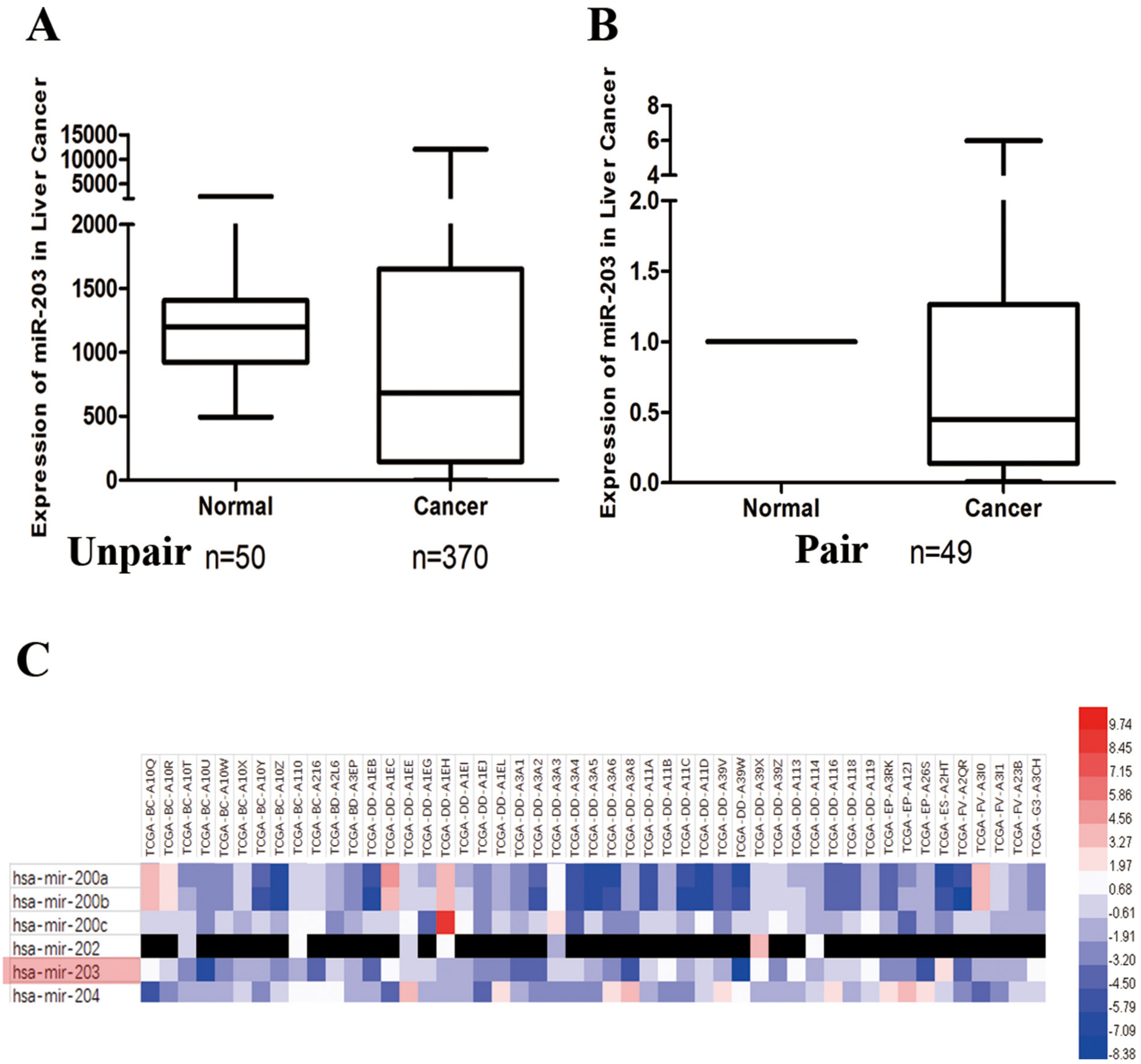

Supplementary Figure S4: The expression of miR-203 in hepatocarcinoma tissues. A-C. The expression of miR-203 in paired or unpaired HCC tissues using the assay of TCGA database (*: $P < 0.05$).

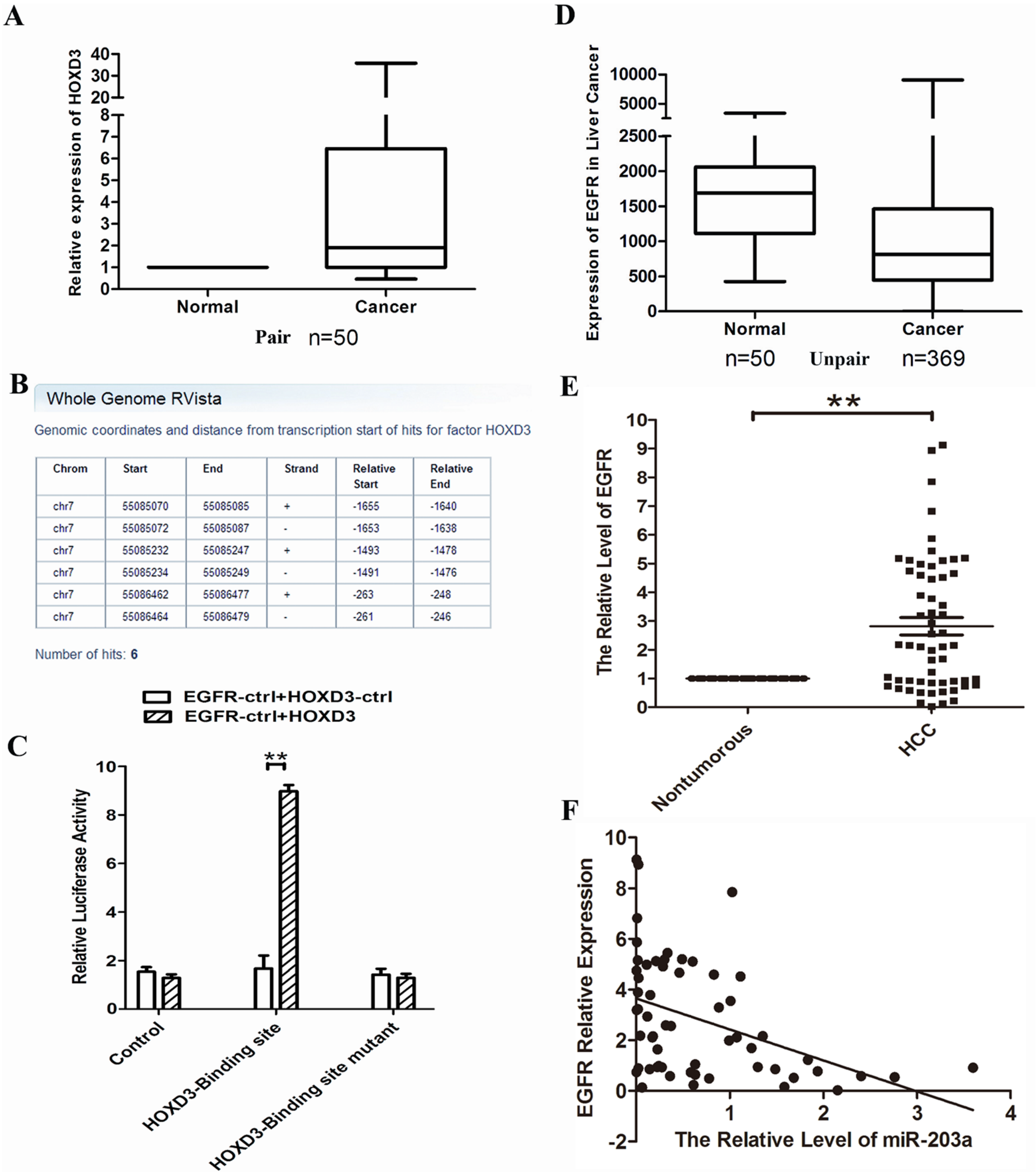

Supplementary Figure S5: MiR-203a inhibits tumor proliferation by targeting HOXD3 through the EGFR signal pathway in hepatocellular carcinoma. A. The expression of HOXD3 in paired HCC tissues using the assay of TCGA database. **B-C.** The interaction of EGFR with HOXD3 was shown using the UCSC genome browser VISTA tool and luciferase assays. **D.** The expression of EGFR in unpaired HCC tissues using the assay of TCGA database. **E.** qRT-PCR was performed to examine EGFR expression in 58 paired human hepatocellular carcinoma tissues and their adjacent nontumor tissues. **F.** There is an inverse correlation between EGFR and miR-203a expression in HCC tissues (**: $P < 0.01$).
